# Dysbiosis in pregnant mice induced by transfer of human vaginal microbiota followed by reversal of pathological changes in the uterus and placenta via progesterone treatment

**DOI:** 10.1186/s12884-024-06595-9

**Published:** 2024-06-14

**Authors:** Gulin Ozcan, Zeynep Gülçe Tanyolaç Talay, Erxiati Paerhati, Ozgur Can Eren, Nilhan Coskun, Deniz Sahin, Iman Alnajjar, Ozgur Albayrak, Attila Gursoy, Ozlem Keskin, Ebru Celik, Fusun Can

**Affiliations:** 1 Koç University İşBank Research Center for Infectious Diseases (KUISCID), Istanbul, Turkey; 2 Division of Gastroenterology and Hepatology, Department of Medicine, University of Illinois at Chicago, Chicago, IL USA; 3 Graduate School of Health Sciences, Koç University School of Medicine, Istanbul, Turkey; 4 College of Engineering, Koç University, Istanbul, Turkey; 5 Department of Pathology, School of Medicine, Koç University, Istanbul, Turkey; 6 Translational Medicine Research Center, Experimental Animals Laboratory, Embryo Research Laboratory, Koç University, Istanbul, Turkey; 7 School of Medicine, Koç University, Istanbul, Turkey; 8 Koç University Hospital Research Center for Translational Medicine, Istanbul, Turkey; 9 Department of Obstetrics and Gynecology, School of Medicine, Koç University, Istanbul, Turkey; 10 Department of Medical Microbiology, School of Medicine, Koç University, Istanbul, Turkey

**Keywords:** Vaginal microbiota, Dysbiosis, Pregnancy, Preterm birth, Inflammation, Progesterone, Animal model

## Abstract

**Objective:**

The vaginal microbiota dysbiosis induces inflammation in the uterus that triggers tissue damage and is associated with preterm birth. Progesterone is used to prevent labor in pregnant women at risk of preterm birth. However, the mechanism of action of progesterone still needs to be clarified. We aimed to show the immunomodulatory effect of progesterone on the inflammation of uterine tissue triggered by dysbiotic vaginal microbiota in a pregnant mouse model.

**Methods:**

Healthy (*n* = 6) and dysbiotic (*n* = 7) vaginal microbiota samples isolated from pregnant women were transferred to control (*n* = 10) and dysbiotic (*n* = 14) pregnant mouse groups. The dysbiotic microbiota transferred group was treated with 1 mg progesterone (*n* = 7). Flow cytometry and immunohistochemistry analyses were used to evaluate inflammatory processes. Vaginal microbiota samples were analyzed by 16 S rRNA sequencing.

**Results:**

Vaginal exposure to dysbiotic microbiota resulted in macrophage accumulation in the uterus and cellular damage in the placenta. Even though TNF and IL-6 elevations were not significant after dysbiotic microbiota transplantation, progesterone treatment decreased TNF and IL-6 expressions from 49.085 to 31.274% (*p* = 0.0313) and 29.279–21.216% (*p* = 0.0167), respectively. Besides, the macrophage density in the uterus was reduced, and less cellular damage in the placenta was observed.

**Conclusion:**

Analyzing the vaginal microbiota before or during pregnancy may support the decision for initiation of progesterone therapy. Our results also guide the development of new strategies for preventing preterm birth.

**Supplementary Information:**

The online version contains supplementary material available at 10.1186/s12884-024-06595-9.

## Introduction

 Vaginal dysbiosis is caused by the replacement of Lactobacillus species with facultative and anaerobic bacteria such as *Gardnerella vaginalis, Atopobium vaginae*, and *Prevotella species* [1]. Vaginal dysbiosis is associated with preterm delivery and newborn health [2] by increasing the susceptibility of the host to inflammatory and metabolic disorders [3]. Women with a *Lactobacillus crispatus* (CST I) vaginal microbiota in pregnancy have a lower risk of preterm birth than those with a dysbiotic microbiota containing *Gardnerella vaginalis* or *Atopobium vaginae* (CST IV) [4]. CST I and CST IV type microbiota components exhibit different metabolic and immune properties [5].

Microorganisms and their products are recognized by Toll-like receptors (TLRs) expressed in the myometrium and placenta during pregnancy [6]. Activation of TLRs induces the expression of proinflammatory cytokines IL-1β, IL-6, IL-8, and tumor necrosis factor-a (TNF). Dysbiosis-related bacteria *G. vaginalis* and *A. vaginae* induce proinflammatory responses, including IL-1B, IL-6, and TNF. In addition, inflammation by microbial products can trigger tissue damage associated with preterm birth by neutrophil, macrophage, and lymphocyte infiltration [7]. Increased inflammatory molecules in uterine components, including IL-1β, IL-6, IL-8, and TNF, have been noted as markers of PTB [8–10]. In a pregnant mouse model, vaginal colonization with dysbiotic microbiota stimulated inflammatory cytokines in the uterus [11]. Likewise, cervicovaginal *G. vaginalis* colonization showed increased IL-6 expression in the cervix during pregnancy [12].

Progesterone reduces the risk of preterm birth in women with a short cervix [13]. Progesterone maintains the pregnancy with the placenta and decreases cervical inflammation [14]. The mechanism of action of vaginal progesterone regarding the prevention of preterm labor is not clearly known. It has been suggested that it promotes anti-inflammatory responses in the uterus or reduces uterine contractility. When stimulated with lipopolysaccharide or lipoteichoic acid, progesterone prevents IL-6 secretion from peripheral blood mononuclear cells [15]. Currently, the vaginal microbiome changes during progesterone therapy are not well known. In the literature, there is only one observational study that reported the effect of progesterone treatment on vaginal microbiota with no change in the microbiota composition [16].

In this study, we examined the role of dysbiotic microbiota in a pregnant mouse model on pro-inflammatory response and the effect of progesterone following healthy and dysbiotic vaginal microbiota inoculation. To determine how different vaginal microbiota communities influence the inflammatory process, we first optimized a vaginal microbiota transplantation model in mice.

## Methods

### Establishment of a mouse model and vaginal microbiota transplantation

#### Selection of vaginal microbiota samples

Human samples and data were obtained by written informed consent by the ethics committee requirements (2019. 093.IRB2.030). For transplantation to mice, we selected seven dysbiotic vaginal microbiota CST IV (low Lactobacillus ratio and high anaerobic pathogen *Gardnerella vaginalis* or *Atopobium vaginae ratio*) and six normal vaginal microbiota composition CST 1 (90% *Lactobacillus crispatus*) isolated from vaginal swabs of pregnant women. The selection criteria were being pregnant at 18 years old or older and with one viable singleton pregnancy. The exclusion criteria were using antibiotics or antifungals 2 weeks before sample collection, vaginal bleeding and cervical cerclage, and having sexual intercourse 72 h before sample collection. Vaginal swabs were stored at − 80 °C until transplantation to mice.

#### Animals

The animal study was approved by the Koç University Biomedical Research Ethics Committee with the Local Ethics Committee for Animal Experiments (2022.HADYEK.039) and performed by the National Institutes of Health Animal Care and Use Guidelines. C57BL/6j 10-week-old female mice were bred in Koç University experimental animal facility under a 12 h light/day photoperiod 50–80% humidity and 25 C temperature conditions with ad libitum water. Timed pregnancies were established by introducing a male to a cage housing two females. After twenty-four hours, the plug was checked and considered embryonic day 0.5 (E0.5).

#### Vaginal microbiota transplantation

The existing vaginal microbiota was destroyed by oral administration of antibiotics with a drinking water (ad libitum) mixture containing ampicillin, gentamycin, metronidazole (1 g/L), 3% sucrose, and 1% glucose antibiotic mixture for 7 days. Starting from the 11th day of pregnancy, vaginal microbiota was transplanted to pregnant mice vaginally for 5 days [17]. Inoculation was achieved by pipetting 20 µL of human vaginal microbiota suspension inocula at the vaginal opening of the mouse in both CSTI and CST IV groups [17, 18].

**Fig. 1:**
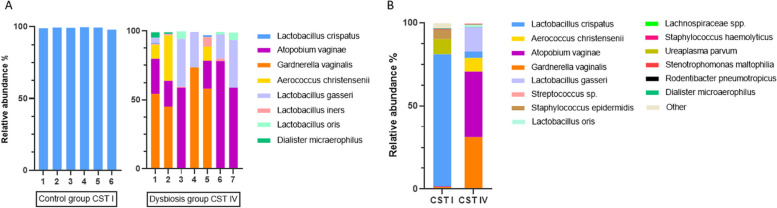
**A** Compositions of vaginal microbiota transplanted into mice. Control group: *Lactobacillus crispatus* dominated CST I type microbiota, Dysbiosis group; *Gardnerella vaginalis* or *Atopobium vaginae* dominated CST IV type microbiota. **B **The relative abundance of the bacterial species was detected in the mice vagina on 18.5 days of pregnancy in CST I and CST IV groups

Six CST I *Lactobacillus crispatus-dominated* samples were selected for transplantation to the mice control group, and seven CST IV *Gardnerella vaginalis* and *Atopobium vaginae-dominated* samples were selected for transplantation to the mice dysbiosis group (Fig. 1A).

#### Progesterone treatment

 After the transfer of the vaginal microbiota, one of the paired pregnant mice from the control group (*n* = 4) and dysbiotic group (*n* = 7) were treated with 17-hydroxyprogesterone caproate intraperitoneally (1 mg) twice in total, one day apart and the other mouse was followed without progesterone [19, 20]. To control the microbiota replacement and the effect of progesterone on microbiota, 16 S rRNA sequencing was performed on vaginal samples collected before the cesarean section at 18.5 days of pregnancy. Preterm birth is defined as delivery before E18.5 [21]. The placenta, cervix, uterus, and peyer patches were collected from each dam.

### Monitoring of inflammatory processes

#### Flow cytometry

For lymphocyte isolation, the uterus and peyer patches were cut into small pieces and homogenized with MACS™ cell dissociation kit. Cells were incubated with a cell activation cocktail with Brefeldin A (BioLegend) for 4 h at 37 °C. After the incubation, cells were stained with viability dye (1:500 in PBS) and incubated for 20 min. Then, 50 µL FACS buffer per well of master mix was prepared containing pre-titrated amounts of the antibodies CD69, CD25, CD19, CD3, and CD4, plus 5 µl/mL of α-CD16/32-Ab (clone 2.4G2). The master mix and the cells were incubated for 20 min in the dark. Stained cells were fixed and permeabilized with BD Cytofix/Cytoperm Fixation/Permeabilization and BD Transcription Factor buffer set. Intracellular staining was performed with the RORgt and cytokines IL-6, IL-17, IL-22, TNF, and IL-1B overnight at 4 °C.

Lymphocytes were identified as SSC-A vs. FSC-A. Doublets were excluded with FSC-W vs. FSC-A and SSC-W vs. SSC-A gating. Live lymphocytes were negative for the live/dead marker. Th17 cells were identified as CD19^−^/CD3CD4^+^ cells. Gating of RORγt^+^ cells vs. IL-17^+^ cells and IL-22^+^ cells. Th1 cells were identified as CD19^−^/CD3^+^CD4^+^ cells. Gating of RORγt^−^ cells vs. IL-1β^+^ cells and IL-6 and TNF^+^ cells. Samples were run using Attune Flow cytometer (Attune NxT Flow Cytometer). Analysis was performed with FlowCo Software.

#### Immunohistochemistry

Cervix, uterus, and placenta tissues from mice were embedded in paraffin 4–6 μm sections. Uterus sections from controls (*n* = 10) and the dysbiotic group (*n* = 14) were incubated with macrophage marker anti-Iba1 antibody (Abcam Cambridge, UK, ab178846), diluted in PBS 1/4000, for 45 min at room temperature. Then, sections were incubated with a secondary antibody using a Mouse and Rabbit-specific detection kit (Abcam Cambridge, UK, ab236466). Counterstains were done using Mayer hematoxylin. Histologic examination was performed. The pathophysiology score was done based on the presence or absence of placental cellular damage as follows: 0 = absent/barely, 1 = occasional/mild, 2 = moderate/high as described (Gilbert, N.M et al.,2021, AJOG) [22].

### DNA isolation and 16 S rRNA sequencing

DNA isolations were performed using the Qiagen DNeasy PowerSoil Kit (Qiagen). Library preparation was performed using QIAseq 16 S/ITS Panel Kit (Qiagen) for sequencing the V1 − V9 region of the 16 S rRNA gene. Library quantification was done using the QIAseq Library Quant Assay (Qiagen) kit with Applied Biosystems QuantStudio 7 Flex Real-Time PCR (Applied Biosystems Inc.). Sequencing was performed with the Illumina MiSeq platform using the MiSeq v3 Reagent Kit (Illumina). All fastq data of the 16 S rRNA sequencing are uploaded to Zenodo database. 10.5281/zenodo.11211477, https://zenodo.org/records/11211477.

### Bioinformatics

FASTQ files were demultiplexed by the different regions using the module in the GeneGlobe Data Analysis Center (https://geneglobe.qiagen.com/tr/analyze). The resulting paired-end FASTQ files containing V1 − V2 region sequences were used to profile the microbiota of the samples with Mothur (v.1.45.3). High-quality sequences were aligned with SILVA bacterial reference database (v.138.1)0.14 Chimeric sequences were removed using the VSEARCH program embedded in the Mothur. Then, the sequences were assigned with taxonomic annotation using the Wang approach implemented in the Mothur. Silva (v.138.1) was used as the reference database for the assignment. Finally, sequences with no more than 3% dissimilarity were clustered into one Operational Taxonomic Unit for the analysis of diversity and composition. The classification of all vaginal trimester samples was identified based on the Community State Types [23] (VaginaL community state type nearest centroId classifier).

### Statistical analysis

The alpha diversity, beta diversity, and vaginal microbiota composition of pregnant women were analyzed. Alpha diversity indices were calculated by the summary. Single command was embedded in the Mothur. Beta-diversity was defined using the Bray − Curtis distance and generated using the dis. shared command in the Mothur. The evaluation of differences in the alpha diversity metrics and microbiota composition was performed by Wilcoxon signed‐rank test using Python 3.7. The significance of group dissimilarity based on the Bray − Curtis distance matrix was evaluated by the analysis of molecular variance (AMOVA) test using Python. For comparison of flow cytometry, results between control and dysbiosis groups were calculated with the Mann-Whitney test and Wilcoxon signed‐rank test. Statistical significance was set as *p* < 0.05. Statistical data were visualized with GraphPad Prism 8.0.2. The animal sample size was calculated using the Resource Equation formula [24].

## Results

 In the dysbiotic microbiota received group, fetal intrauterine death (pups born death, smaller than normal) and three abortus (immature fetal tissue) were seen in the two matched pairs. Among them, preterm births (28.5%) were seen in the two dysbiotic microbiota-transferred CST IV groups. There was no adverse fetal outcome in the normal microbiota-received mice group (Table 1).

**Table 1:**
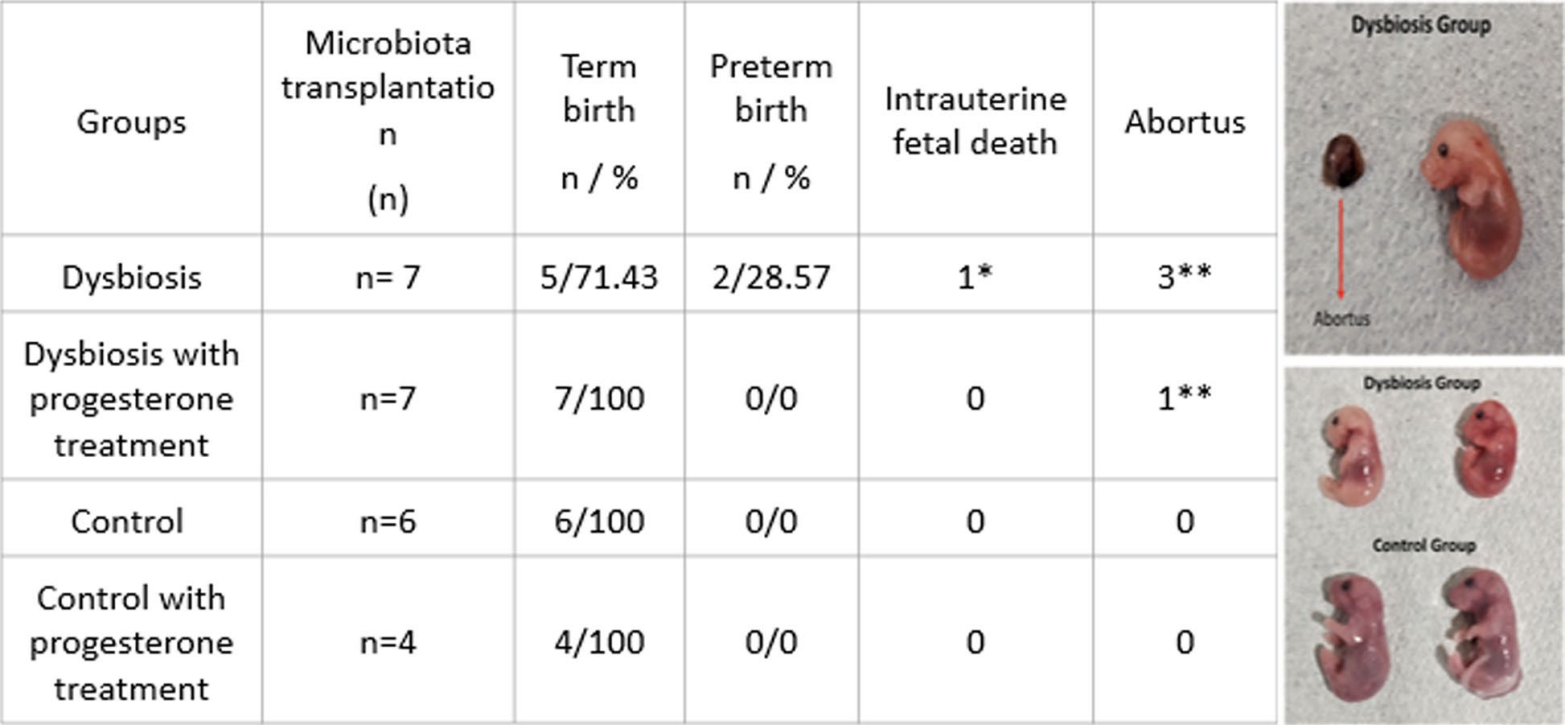
Mouse model pregnancy outcomes

*One intrauterine fetal death was seen in 48 pups of the dysbiosis group

**Three abortus were seen in 48 pups of the dysbiosis group

**One abortus was seen in 50 pups of the dysbiosis group with progesterone treatment

### Confirmation of vaginal microbiota transplantation to mice

In the 16 S rRNA analysis of mouse vaginal samples on day 18.5 of transfer, we observed that the microbiota was in a similar ratio with the donors in all mouse groups. We also confirmed the depletion of the mouse microbiota. We detected low rates (<%1) of mouse microbiota-related species such as *Rodentibacter pneumotropicus* (Fig. 1B).

### The effect of progesterone treatment on dysbiotic microbiota composition

**Fig. 2:**
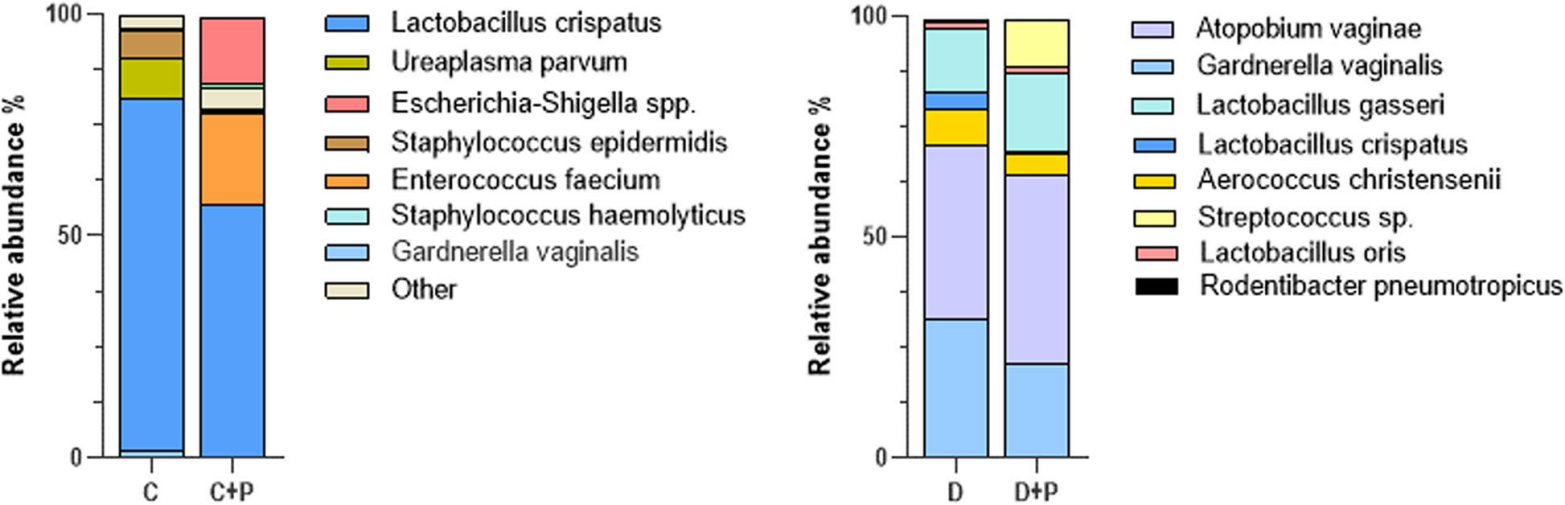
Comparison of dysbiotic microbiota transferred mice vaginal microbiota species proportion before and after progesterone treatment. C: Healthy vaginal microbiota (CST-I) transferred mice, C + P: Healthy vaginal microbiota (CST-I) transferred mice after progesterone treatment, D: Dysbiotic vaginal microbiota (CST-IV) transferred mice, D + P: Dysbiotic vaginal microbiota (CST-IV) transferred mice after progesterone treatment

There was no significant change in microbiota composition after the progesterone treatment (Shannon index, *P* = 0.63; Chao index *P* = 0.14). The proportion of human microbiota-related species including *Atopobium vaginae* and *Lactobacillus gasseri* slightly increased, while *Gardnerella vaginae* and *Aeroccous christensenii* decreased after treatment (Fig. 2). In the control group, the proportion of *Lactabacillus crispatus* decreased, and *Enterococcus faecium*, *Escherichia-Shigella spp.* increased after progesterone treatment.

### Inflammation in mice uterine tissue and peyer patches

**Fig. 3:**
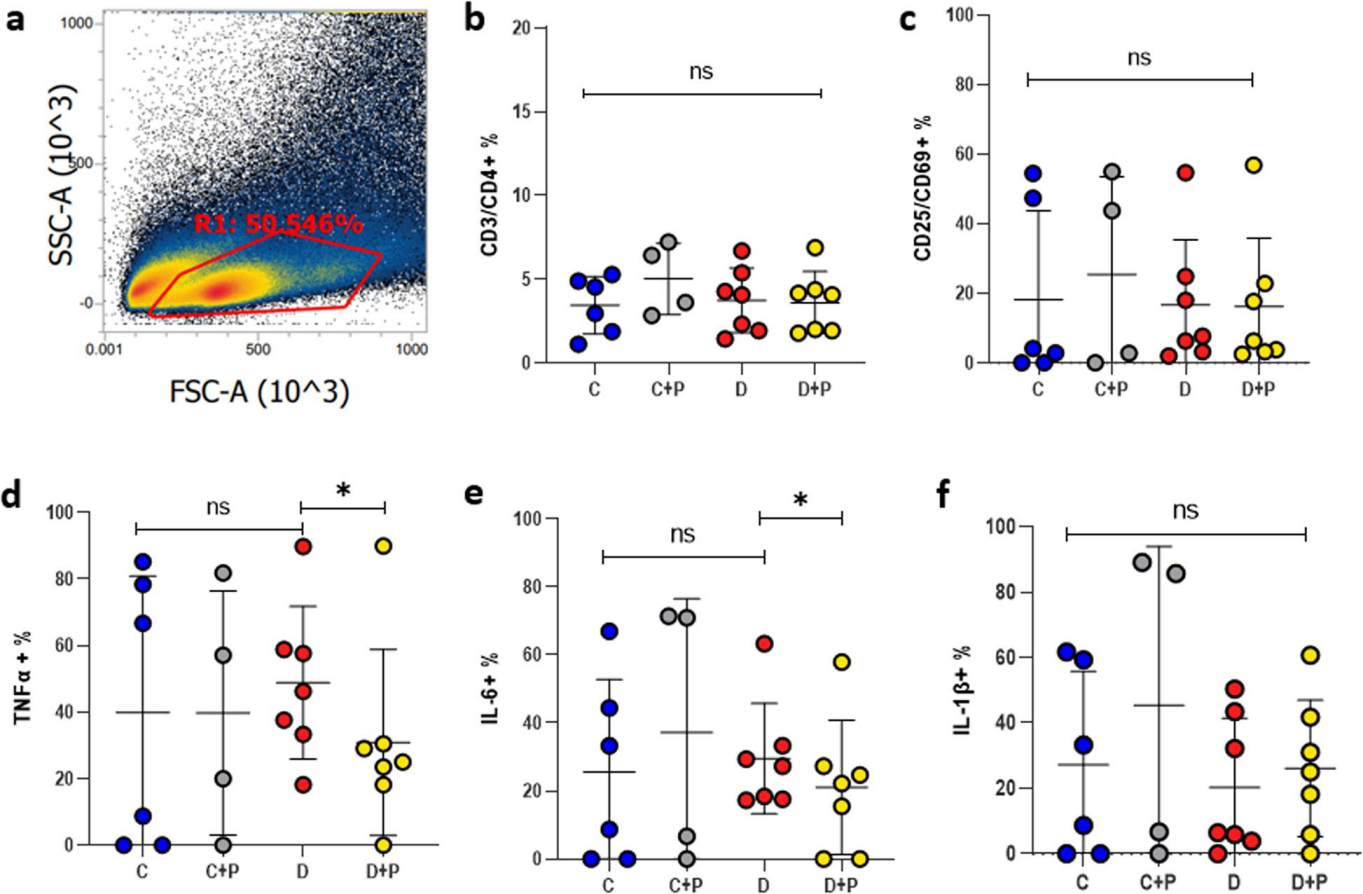
Lymphocyte cells isolated from the uterine tissue (**a**). Effect of human dysbiotic vaginal microbiota and progesterone treatment on the production of CD3/CD4 + immune cells in uterine tissues (**b**), CD25/CD69 + cells (**c**), tumor necrosis factor-alpha (TNF) (**d**), pro-inflammatory cytokines interleukin-6 (IL-6) (**e**), interleukin-1β (IL-1β) (**f**). C (Control Group); healthy vaginal microbiota transplanted mice (*n* = 6), D (Dysbiosis group); vaginal dysbiosis microbiota transplanted mice (*n* = 7), (C + P); Control group with progesterone treatment; mice receiving 1 mg/ml progesterone (*n* = 4), (D + P); Dysbiosis group with progesterone treatment; mice receiving 1 mg/ml progesterone (*n* = 7)

There was no significant difference in the IL-6+ (25.460% vs. 29.279%; *p* = 0.756) and IL-1β+ (26.5% vs. 20.5%; *p* = 0.727) expression levels between dysbiotic and normal microbiota received groups (Fig. 3e, f). TNF + cell ratio was higher but not significantly different in the dysbiosis group compared to the control group (48.546% vs. 39.910%; *p* = 0.6296) (Fig. 3d). CD3 + CD4 + T cells (3.39% vs. 3.84%; *p* = 0.629) and CD25 + CD69+ (18.561% vs. 16.835%; *p* = 0.42) cells were similar between the two groups (Fig. 3b, c).

After progesterone treatment, TNF significantly decreased from 49.085 to 31.274% (*p* = 0.0313) and the IL-6 + cells ratio significantly decreased from 29.279 to 21.216% (*p* = 0.0167) in the dysbiosis group, respectively (Fig. 3d, e). In six out of seven mice, TNF + levels were significantly reduced after treatment. Similarly, in all mice of the dysbiosis group, IL-6 levels decreased after treatment.

**Fig. 4:**
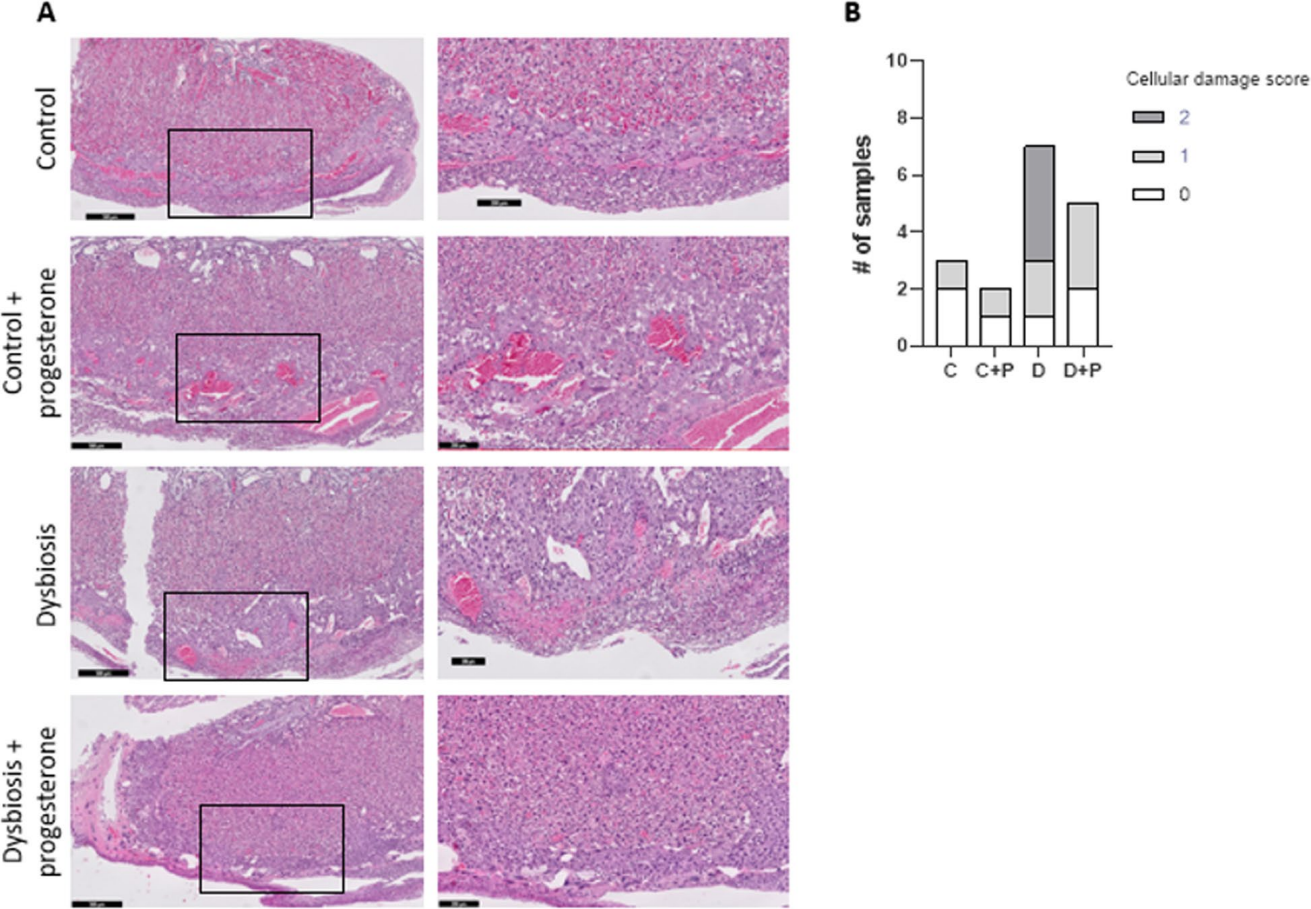
H&E-stained formalin-fixed mouse placental section (**A**) scoring of the placental cellular damage examination **C**; Control group *n* = 3, C + P; Progesterone treated control group *n* = 2, D; Dysbiosis group *n* = 7, D + P; Progesterone treated dysbiosis group *n* = 5 (**B**). Cellular damage score; 0: absent/barely, 1: occasional/mild, 2: moderate/high

In the immunohistochemical evaluation of placenta sections, 5 samples from the control group and 12 samples from the dysbiosis group were evaluated. 42.9% of the dysbiotic microbiota transferred group had moderate/high cell damage which decreased to 1 or 2 (absent/rarely) after progesterone treatment (Fig. 4B). There was no apparent cell damage in the healthy vaginal microbiota transferred control group (Fig. 4A).

**Fig. 5:**
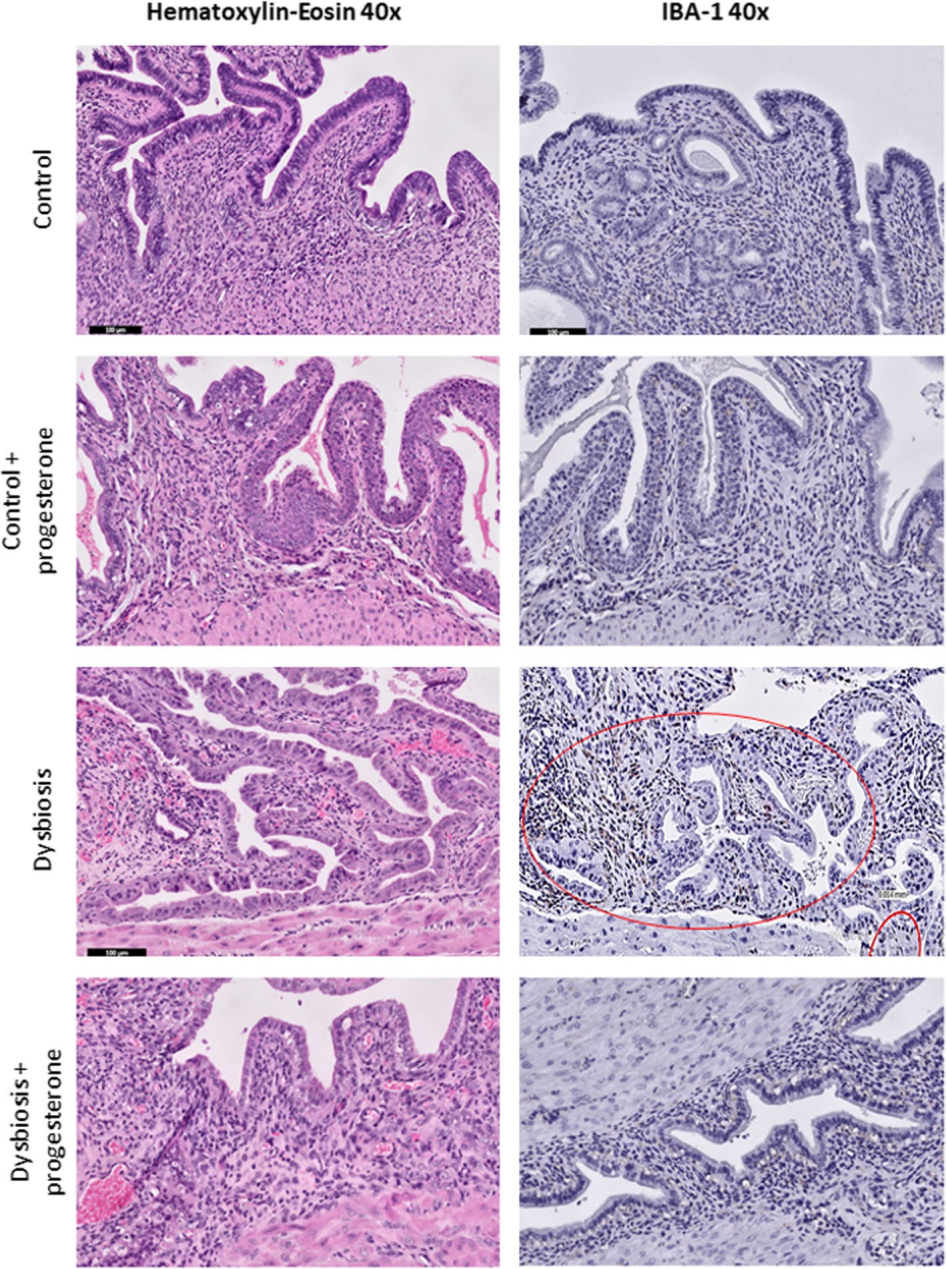
Evaluation of macrophage infiltration with IBA-1 antibody in mouse uterus sections. Hematoxylin-eosin sections (left panel) demonstrated dysbiotic mice harboring increased macrophage infiltration compared to control mice (with and without progesterone). Under progesterone treatment, dysbiotic mice had fewer macrophages. The findings were also seen with anti-IBA-1 staining (right panel). Macrophage accumulation was marked in the red circle area

Hematoxylin-eosin sections and anti-IBA-1 staining demonstrated dysbiotic mice harboring increased macrophage infiltration compared to control mice (with and without progesterone). Under progesterone treatment, dysbiotic mice had fewer macrophages (Fig. 5).

In the Peyer patches, there was no significant difference between the control group (*n* = 5) and the dysbiosis group (*n* = 5) in the production of IL-17 (16.456% vs. 18.565%; *p* = 0.42), CD3 + CD4+ (17.698% vs. 15.971; *p* = 0.841) and CD25 + CD69+ (24.143% vs. 18.331%; *p* = 0.285) T cells. IL-22 (17.620% vs. 0.92%; *p* = 0.587) was lower in the dysbiosis group than in the control group (Supplement Fig. 1). After the progesterone treatment, CD3 + CD4 + T cells decreased from 16.403 to 10.791% in the dysbiosis group. There was no significant change in the CD25 + CD69 + T cell and IL-17 + and IL-22 + production after the progesterone treatment (Supplementary Fig. 1).

## Discussion

In this study, dysbiotic microbiota transplantation did not cause a significant increase in the expression of TNF and IL-6 in the uterus of pregnant mice. However, the accumulation of macrophages in the uterus and moderate/severe cell damage in the placenta might result in inflammation in the uterus. Progesterone treatment suppressed the expression of TNF and IL-6 responses and macrophage density in the uterus, along with lowering cell damage in the placenta.

Premature birth is a major cause of perinatal mortality and morbidity worldwide, and an estimated 13.4 million babies were born prematurely in 2020 [25]. It greatly affects developmental disabilities such as neurodevelopmental abnormalities, immature organ systems, behavioral disorders, and quality of life [26]. Various reasons lead to preterm birth, particularly intrauterine infections are considered one of the main causes of the reasons because they trigger inflammation in the uterus.

Increasing levels of infection-mediated pro-inflammatory cytokines, including TNF, IL-1β, IL-6, and IL-2, have been linked to chorioamnionitis and adverse pregnancy outcomes [27, 28]. Maternal-fetal interaction has mechanisms that lower the effects of this pro-inflammatory response and maintain pregnancy [29]. Therefore, we investigated the expression of TNF, IL-1B, and IL-6 in the uterus for healthy (*L. crispatus* dominated) and dysbiotic microbiota-transferred pregnant mice. There was no significant increase in TNF and IL-6 levels after transplantation of the dysbiotic microbiota.

In the immune histochemistry examination, macrophage density in the uterus and placental cellular damage were higher in the dysbiotic group compared to controls. The dysbiotic microbiota composition is important in the level of inflammatory response. *Gardnerella vaginalis* riched microbiota (CST IV) showed higher proinflammatory cytokine levels compared to *Lactobacillus crispatus-dominated* microbiota (CST I) in the uterine tissue [30] and PTB [7]. In contrast, the *G. vaginalis* and *P. bivia* microbiota had caused no histologic inflammation in vaginal tissue [31].

We observed premature birth and abortion in the two dysbiotic microbiota-transferred mice. The inflammation stimulatory activity of *A. vaginae* was shown in the human vaginal epithelial cell model [32]. It is argued that *A. vaginae* may be a robust immune response stimulator and contributes to adverse birth outcomes like *G.vaginalis.* Furthermore, *A. vaginae and G. vaginalis* immune mediator profiles are different from *P. bivia, L. iners* and *L. crispatus* [33]. Our results supported the importance of *A. vaginae and G. vaginalis* in dysbiotic microbiota in triggering inflammation markers. Following the literature, we suggest the pathogenesis of vaginal dysbiosis should be more intensively studied with different types of dysbiotic vaginal microbiota composition [34].

In this study, the IL-6 + cells ratio (from 29.279 to 21.216% (*p* = 0.0167) and TNF (from 49.085 to 31.274% (*p* = 0.0313)) decreased after the progesterone treatment in the dysbiotic vaginal microbiota-induced group. Also, macrophage density and cellular damage were lower after progesterone treatment. Progesterone has been shown to significantly reduce IL-4-induced macrophage expression, IL-8, and Toll-like receptors 2 and 4 triggered by lipopolysaccharides in the cervix and placenta [35, 36]. In a clinical trial, 17α-hydroxyprogesterone caproate treatment was shown to reduce IL-1β but not IL-6, IL-8, and TNF in peripheral blood samples in vitro models of Gram-positive and Gram-negative bacterial infection [15]. However, these data could not clarify the anti-inflammatory mechanism of progesterone in the uterus. Previous studies with *E. coli* and LPS induction have shown that progesterone reduces inflammation in the genital tract. In this study, by the transfer of dysbiotic microbiota, we found a reduction in macrophage accumulation in the uterus and a decrease in cytopathology in the placenta with progesterone treatment along with downregulation in cytokine expressions, but there was no significant difference in the microbiota composition. In the literature, there are only a couple of studies related to the effect of progesterone on vaginal microbiota. One study showed that progestin oral treatment does not significantly change vaginal microbiota [16]. Our results suggested that the preventative effect of progesterone on preterm birth is related to anti-inflammatory activity rather than the modification of bacterial taxa in vaginal microbiota. These results are important to determine the mechanism of action of progesterone in the treatment of dysbiosis and to develop strategies for preventing adverse pregnancy outcomes.

Studies investigating the relationship between *G. vaginalis* infection-associated BV and PTB show controversial results since this bacterium can be found in also healthy women [37]. This suggests that *G. vaginalis* alone is not the only cause of triggering preterm labor; therefore, transfer of pure *G.vaginalis* culture in mouse models for assessment of preterm delivery may not be appropriate.

### Strengths and limitations

The main strength of this study is that in our mouse model, we transferred a complete healthy or dysbiotic vaginal microbiota (rich for *Gardnerella vaginalis, Atopobium vaginae*) obtained from different pregnant patients. The sustainability of microbiota colonization at the time of delivery indicates that our experimental design is an appropriate mouse model for vaginal microbiota studies.

We must acknowledge a few limitations. We aimed to see the impact of different vaginal microbiota compositions on the gut immune response also. In mouse models, it has been suggested that a dysbiotic vaginal microbiota may induce a similar phenotype in the gut [38]. However, studies are limited. In our study, we couldn’t detect any changes and differences in the intestinal immune system between healthy and dysbiotic vaginal microbiota groups. This may be related to vaginal microbiota transfer in our cohort that could cause inadequate bacterial colonization in the gut during the pregnancy period.

## Conclusion

Dysbiotic microbiota induces macrophage accumulation and necrosis in the uterus which can be reversed by progesterone treatment. Progesterone treatment also suppresses the secretion of proinflammatory cytokines, particularly TNF with no significant change in microbiota composition. Analyzing the vaginal microbiota before or during pregnancy may support the decision for initiation of progesterone therapy. Besides, our results may guide new strategies like personalized treatments with novel drugs for preventing preterm birth.

### Supplementary Information

Supplementary Material 1.

## Data Availability

All raw data of the microbiota analysis are uploaded to Zenodo database. https://doi.org/10.5281/zenodo.11211477, https://zenodo.org/records/11211477.

## References

[1] Swidsinski A, Mendling W, Loening-Baucke V, Ladhoff A, Swidsinski S, Hale LP, Lochs H (2005). Adherent biofilms in bacterial vaginosis. Obstet Gynecol.

[2] Hillier SL, Nugent RP, Eschenbach DA, Krohn MA, Gibbs RS, Martin DH, Cotch MF, Edelman R, Pastorek JG, Rao AV, McNellis D (1995). Association between bacterial vaginosis and preterm delivery of a low-birth-weight infant. N Engl J Med.

[3] Petersen C, Round JL (2014). Defining dysbiosis and its influence on host immunity and disease. Cell Microbiol.

[4] Donders GG, Van Calsteren K, Bellen G, Reybrouck R, Van den Bosch T, Riphagen I, Van Lierde S (2009). Predictive value for preterm birth of abnormal vaginal flora, bacterial vaginosis and aerobic vaginitis during the first trimester of pregnancy. BJOG.

[5] Greenbaum S, Greenbaum G, Moran-Gilad J, Weintraub AY (2019). Ecological dynamics of the vaginal microbiome in relation to health and disease. Am J Obstet Gynecol.

[6] Pudney J, He X, Masheeb Z, Kindelberger DW, Kuohung W, Ingalls RR (2016). Differential expression of toll-like receptors in the human placenta across early gestation. Placenta.

[7] Romero R, Gotsch F, Pineles B, Kusanovic JP (2007). Inflammation in pregnancy: its roles in reproductive physiology, obstetrical complications, and fetal injury. Nutr Rev.

[8] Fettweis JM, Serrano MG, Brooks JP, Edwards DJ, Girerd PH, Parikh HI, Huang B, Arodz TJ, Edupuganti L, Glascock AL, Xu J (2019). The vaginal microbiome and preterm birth. Nat Med.

[9] Kemp MW, Saito M, Newnham JP, Nitsos I, Okamura K, Kallapur SG (2010). Preterm birth, infection, and inflammation advances from the study of animal models. Reproductive Sci.

[10] Taylor BD, Holzman CB, Fichorova RN, Tian Y, Jones NM, Fu W, Senagore PK (2013). Inflammation biomarkers in vaginal fluid and preterm delivery. Hum Reprod.

[11] Wolfarth AA, Smith TM, VanInsberghe D, Dunlop AL, Neish AS, Corwin EJ, Jones RM (2020). A human microbiota-associated murine model for assessing the impact of the vaginal microbiota on pregnancy outcomes. Front Cell Infect Microbiol.

[12] Sierra LJ, Brown AG, Barilá GO, Anton L, Barnum CE, Shetye SS, Soslowsky LJ, Elovitz MA (2018). Colonization of the cervicovaginal space with Gardnerella vaginalis leads to local inflammation and cervical remodeling in pregnant mice. PLoS ONE.

[13] Fonseca EB, Celik E, Parra M, Singh M, Nicolaides KH (2007). Progesterone and the risk of preterm birth among women with a short cervix. N Engl J Med.

[14] Larsen B, Hwang J (2011). Progesterone interactions with the cervix: translational implications for term and preterm birth. Infect Dis Obstet Gynecol.

[15] Foglia LM, Ippolito DL, Stallings JD, Zelig CM, Napolitano PG (2010). Intramuscular 17α-hydroxyprogesterone caproate administration attenuates immunoresponsiveness of maternal peripheral blood mononuclear cells. Am J Obstet Gynecol.

[16] Kindinger LM, Bennett PR, Lee YS, Marchesi JR, Smith A, Cacciatore S, Holmes E, Nicholson JK, Teoh TG, MacIntyre DA (2017). The interaction between vaginal microbiota, cervical length, and vaginal progesterone treatment for preterm birth risk. Microbiome.

[17] Chen T, Xia C, Hu H, Wang H, Tan B, Tian P, Zhao X, Wang L, Han Y, Deng KY, Wei H (2021). Dysbiosis of the rat vagina is efficiently rescued by vaginal microbiota transplantation or probiotic combination. Int J Antimicrob Agents.

[18] Jašarević E, Hill EM, Kane PJ, Rutt L, Gyles T, Folts L, Rock KD, Howard CD, Morrison KE, Ravel J, Bale TL (2021). The composition of human vaginal microbiota transferred at birth affects offspring health in a mouse model. Nat Commun.

[19] Novak CM, Ozen M, McLane M, Alqutub S, Lee JY, Lei J, Burd I (2018). Progesterone improves perinatal neuromotor outcomes in a mouse model of intrauterine inflammation via immunomodulation of the placenta. Am J Reprod Immunol.

[20] Smithberg M (1958). Attempts to induce and maintain pregnancy in prepuberal mice following treatment with 17α-Hydroxyprogesterone 17‐n‐CAPROATE. Ann N Y Acad Sci.

[21] Spencer NR, Radnaa E, Baljinnyam T, Kechichian T, Tantengco OA, Bonney E, Kammala AK, Sheller-Miller S, Menon R (2021). Development of a mouse model of ascending infection and preterm birth. PLoS One.

[22] Gilbert NM, Foster LR, Cao B, Yin Y, Mysorekar IU, Lewis AL (2021). Gardnerella vaginalis promotes group B Streptococcus vaginal colonization, enabling ascending uteroplacental infection in pregnant mice. Am J Obstet Gynecol.

[23] France MT, Ma B, Gajer P, Brown S, Humphrys MS, Holm JB, Waetjen LE, Brotman RM, Ravel J (2020). VALENCIA: a nearest centroid classification method for vaginal microbial communities based on composition. Microbiome.

[24] Arifin WN, Zahiruddin WM (2017). Sample size calculation in animal studies using resource equation approach. Malaysian J Med Sci.

[25] Ohuma EO, Moller AB, Bradley E, Chakwera S, Hussain-Alkhateeb L, Lewin A, Okwaraji YB, Mahanani WR, Johansson EW, Lavin T, Fernandez DE (2023). National, regional, and global estimates of preterm birth in 2020, with trends from 2010: a systematic analysis. Lancet.

[26] Ward RM, Beachy JC (2003). Neonatal complications following preterm birth. BJOG.

[27] Holst RM, Laurini R, Jacobsson B, Samuelsson E, Sävman K, Doverhag C, Wennerholm UB, Hagberg H (2007). Expression of cytokines and chemokines in cervical and amniotic fluid: relationship to histological chorioamnionitis. J Maternal-Fetal Neonatal Med.

[28] Revello R, Alcaide MJ, Dudzik D, Abehsera D, Bartha JL (2016). Differential amniotic fluid cytokine profile in women with chorioamnionitis with and without funisitis. J Maternal-Fetal Neonatal Med.

[29] Sadowsky DW, Adams KM, Gravett MG, Witkin SS, Novy MJ (2006). Preterm labor is induced by intraamniotic infusions of interleukin-1β and tumor necrosis factor-α but not by interleukin-6 or interleukin-8 in a nonhuman primate model. Am J Obstet Gynecol.

[30] Hardy L, Jespers V, Abdellati S, De Baetselier I, Mwambarangwe L, Musengamana V, Van De Wijgert J, Vaneechoutte M, Crucitti T (2016). A fruitful alliance: the synergy between Atopobium vaginae and Gardnerella vaginalis in bacterial vaginosis-associated biofilm. Sex Transm Infect.

[31] Gilbert NM, Lewis WG, Li G, Sojka DK, Lubin JB, Lewis AL (2019). Gardnerella vaginalis and Prevotella bivia trigger distinct and overlapping phenotypes in a mouse model of bacterial vaginosis. J Infect Dis.

[32] Doerflinger SY, Throop AL, Herbst-Kralovetz MM (2014). Bacteria in the vaginal microbiome alter the innate immune response and barrier properties of the human vaginal epithelia in a species-specific manner. J Infect Dis.

[33] Ramsey PS, Lyon MD, Goepfert AR, Cliver S, Schwebke J, Andrews WW, Goldenberg RL, Hauth JC (2005). Use of vaginal polymorphonuclear to epithelial cell ratios for the prediction of preterm birth. Obstet Gynecol.

[34] van de Wijgert JH, Jespers V (2017). The global health impact of vaginal dysbiosis. Res Microbiol.

[35] Elovitz MA, Mrinalini C (2005). Can medroxyprogesterone acetate alter toll-like receptor expression in a mouse model of intrauterine inflammation?. Am J Obstet Gynecol.

[36] Menzies FM, Henriquez FL, Alexander J, Roberts CW (2011). Selective inhibition and augmentation of alternative macrophage activation by progesterone. Immunology.

[37] Srinivasan S, Liu C, Mitchell CM, Fiedler TL, Thomas KK, Agnew KJ, Marrazzo JM, Fredricks DN (2010). Temporal variability of human vaginal bacteria and relationship with bacterial vaginosis. PLoS One.

[38] Amabebe E, Anumba DO (2020). Female gut and genital tract microbiota-induced crosstalk and differential effects of short-chain fatty acids on immune sequelae. Front Immunol.

