# Supplementary figures and images for: Dysbiosis in pregnant mice induced by transfer of human vaginal microbiota followed by reversal of pathological changes in the uterus and placenta via progesterone treatment
Source: BMC Pregnancy Childbirth. 2024 Jun 14;24:427. doi: 10.1186/s12884-024-06595-9 (PMC11177491; doi:10.1186/s12884-024-06595-9)

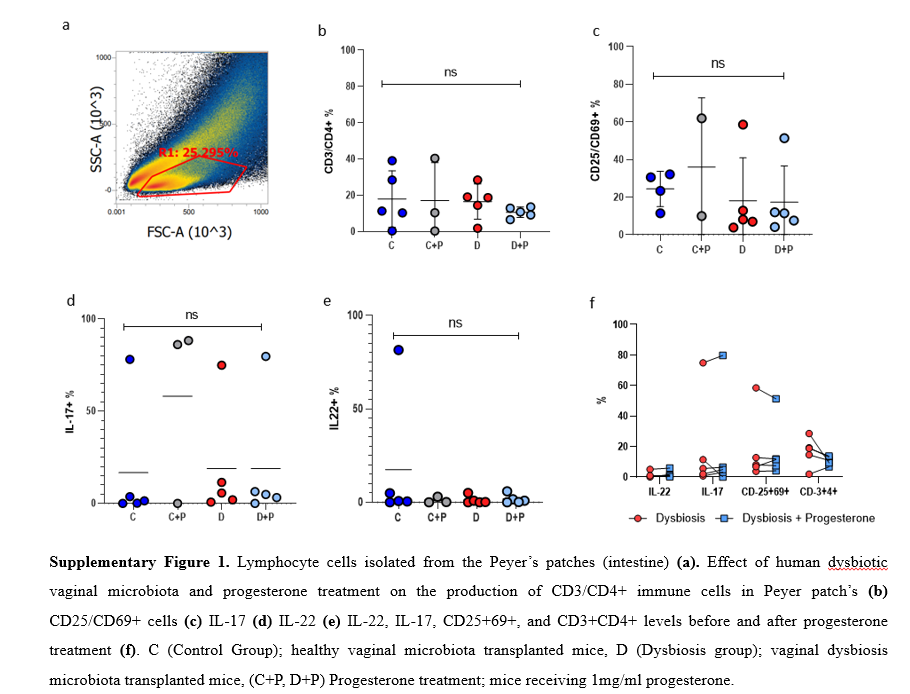

Supplement: Supplementary file 1 — Supplementary Material 1. [file 12884_2024_6595_MOESM1_ESM.png]
